# CRP (C-Reactive Protein) Revisited: An Old Yet New Biomarker of Acute and Chronic Inflammation

**DOI:** 10.3390/cells15110998

**Published:** 2026-05-29

**Authors:** Masayuki Nagasawa

**Affiliations:** 1 Department of Pediatrics and Developmental Biology, Institute of Science Tokyo, 1-5-45 Bunkyo-Ku, Tokyo 113-8519, Japan; mnagasawa.ped@tmd.ac.jp; Tel.: +81-0480-93-8556; 2 Ohbayashi Medical Clinic, 656-1 Sendano, Shiraoka-city 349-0215, Saitama, Japan

**Keywords:** C-reactive protein, inflammation, C-polysaccharide, IL-6, single-nucleotide polymorphism, biomarker, innate immunity

## Abstract

C-reactive protein (CRP) was discovered as a protein that reacts with C-polysaccharides to form precipitates in the serum of patients with pneumococcal infection. Subsequently, it was found to increase in the serum of patients with bacterial infections and rheumatic diseases, and it has since been widely recognized as a nonspecific biomarker of acute inflammation and utilized in clinical medicine. Meanwhile, CRP-like proteins are also present in the hemolymph of horseshoe crabs, and it has become clear that these proteins have long played a crucial role in the humoral innate immune response against foreign microorganisms. In recent years, advances in molecular analysis have revealed the details of the complex biological functions performed by CRP. Furthermore, with the development of highly sensitive CRP measurement methods, its importance as a biomarker is gaining attention not only in acute inflammatory diseases but also in chronic inflammatory diseases such as cardiovascular disease, diabetes, cancer and neurological disorders. New treatment strategies targeting CRP, based on recent findings, are also being explored.

## 1. Introduction

In 1930, Tillett and Francis found a serum factor from patients with pneumococcal pneumonia that reacted to form a precipitate with polysaccharide from the cell wall of *Streptococcus pneumoniae* [1]. C-polysaccharides are characterized by a tetra-saccharide backbone linked by ribitol phosphate groups and modified with phosphorylcholine. It was named “C-reactive protein” later, because it reacted with the third polysaccharide component to be isolated, in reference to the third letter of the alphabet. CRP has the property of binding to phosphocholine in a calcium-dependent manner. CRP has been detected not only in the serum of patients with pneumococcal pneumonia but also in the serum of patients with rheumatic fever and other bacterial infections [2]. Over many years, CRP has been recognized and used as a nonspecific diagnostic biomarker of inflammation. It is only very recently that details of its biological functions have emerged regarding the active role CRP plays as an inflammatory mediator in the innate immune system [3]. Today, CRP is gaining attention not only as a biomarker for acute inflammatory conditions such as bacterial infections and rheumatic diseases, but also as a biomarker for chronic inflammatory conditions such as cardiovascular disease, diabetes, cancer, and neurological disorders. Furthermore, as molecular analytical methods have elucidated the mechanism of action of CRP in innate immunity, it has gained more attentions as a potential target for immunomodulatory therapeutic interventions. A literature search was conducted up to January 2026 through the following databases; PubMed, Scopus, Google Scholar, MEDLINE, and J-Stage, using the following keywords, “CRP”, “innate immunity”, inflammation”, “biomarker”, “cytokine” and “immune regulation”. Furthermore, to explore specific issues in greater depth, I expanded the literature search by combining keywords such as “phylogeny”, “genetic polymorphisms,” “cardiovascular diseases,” and “metabolic diseases.” This article provides an overview of CRP to help readers gain a broader understanding of acute and chronic inflammatory responses from a new perspective.

## 2. Tertiary Structure of CRP and Its Transformation to Dissociation

**Figure 1:**
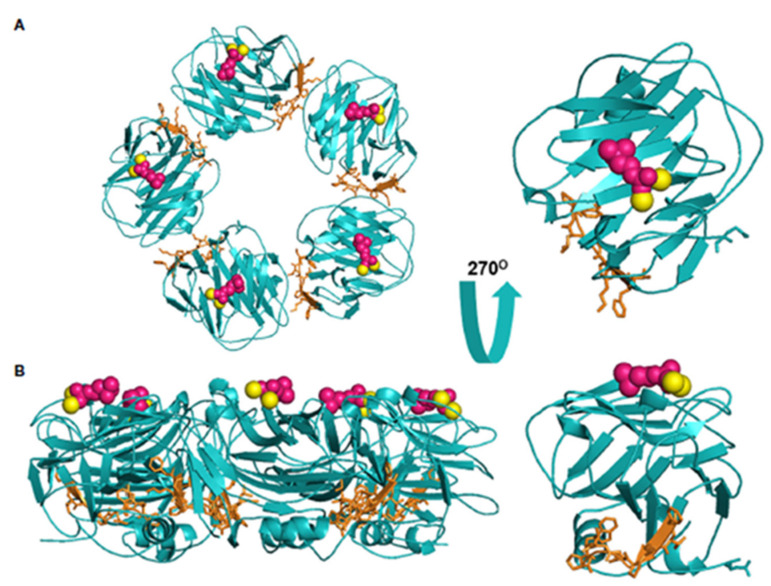
**Structure of pentameric CRP (pCRP) and monomeric CRP (mCRP).** CRP is stable in the presence of two calcium ions (Ca: yellow spheres), forms a pentamer, and binds to phosphocholine (PC: pink spheres). Upon binding to PC, the tertiary structure changes, and a neoepitope (CRP C-terminal aa199–206: orange) is exposed. (The transitional form pCRP* is not shown in the figure above). Subsequently, the pentamer dissociates into monomers. pCRP* and mCRP bind to complement C1q, but the binding site is on the opposite side of the site where they bind to PC [12,13]. The figure reproduced with permission from Olson ME et al., Frontiers in Immunology; published by frontiers, 2023 [22]. (**A**) provides a top-down view, and (**B**) a side profile of pCRP and pCRP monomer.

The human CRP gene is located on chromosome 1q23 and consists of two exons. The first exon encodes the leader peptide and the first two amino acid residues of the mature protein, while the second exon encodes the remaining 204 amino acids. Human CRP consists of 206 amino acids and contains cysteine residues at positions 36 and 97, which contribute to disulfide bonds. The 23-kDa protein exists as a 115-kDa pentamer via noncovalent interactions, forming a central core and adopting a symmetrical disc-like conformation [3]. This structure is called pentameric CRP (pCRP) and exists in a soluble state in plasma. CRP forms a stable pentamer by binding to two Ca^2+^ ions (Figure 1) [4]. When pCRP binds to PC in bacterial or cellular membranes, a transient structural change occurs (referred to as pCRP*), and a new neoepitope (aa199–206) appears while maintaining the pentameric structure (In pCRP, the neoepitope is hidden within the pentameric structure) [5,6,7]. Because neoepitopes are hydrophobic, they form nonspecific interactions with lipid rafts on the cell membrane [8], and direct binding to FcγRIIIa (CD16) has also been suggested [9]. Subsequently, pCRP* is further separated into monomeric CRP (mCRP) [10,11]. It is reported that pCRP is completely dissociated into mCRPs within 24 to 48 h [5]. pCRP* and mCRP bind to complement C1q on the opposite side of their binding sites with PC [12,13]. In vitro, pCRP is converted to mCRP by heat treatment, acid treatment, the addition of chelating agents, or oxidative stress [14]. In vitro, unlike pCRP, mCRP precipitates as aggregates because it is insoluble in plasma. When the S-S bond within mCRP is cleaved, it exhibits even stronger proinflammatory activity [15]. The antibodies used in standard serum CRP assays (latex agglutination method) recognize both pCRP and mCRP; however, pCRP and mCRP can be distinguished by using structure-specific antibodies such as 9C9 and 3H12 [16,17,18,19,20,21]. However, this antibody cannot distinguish between pCRP* and mCRP. The transition from pCRP to pCRP* to mCRP is considered to be unidirectional and irreversible. It has now been established that CRP present in the membrane (lipid rafts) is rich in pCRP*/mCRP isoforms, whereas CRP in the aqueous phase lacks neoepitopes.

## 3. CRP Belongs to the Pentraxin Family

CRP-like proteins are also present in the hemolymph of horseshoe crabs and have long been thought to play a crucial biological role in innate immunity, which mediates responses to foreign microorganisms. CRP-like substances in the hemolymph of arthropods (Anthropoda), echinoderms (Echinodermata), and mollusks (Mollusca) are constantly present but do not respond to stimuli such as infection; in contrast, CRP in vertebrates is induced in response to infection, other environmental factors (such as oxidative stress), and toxins. Furthermore, while CRP is primarily produced in the liver in vertebrates, in invertebrates it is produced by various tissues and organs. Five CRP genes have been identified in Atlantic salmon. These are divided into Group I (CRP/SAP-1a, CRP/SAP-1b, CRP/SAP-1c, CRP/SAP-2) and Group II (CRP/SAP-3). While Group I is found in mammals, Group II is said to be characteristic of fish and amphibians. CRP/SAP-1a is induced by cytokines, but the other four are not.

**Figure 2:**
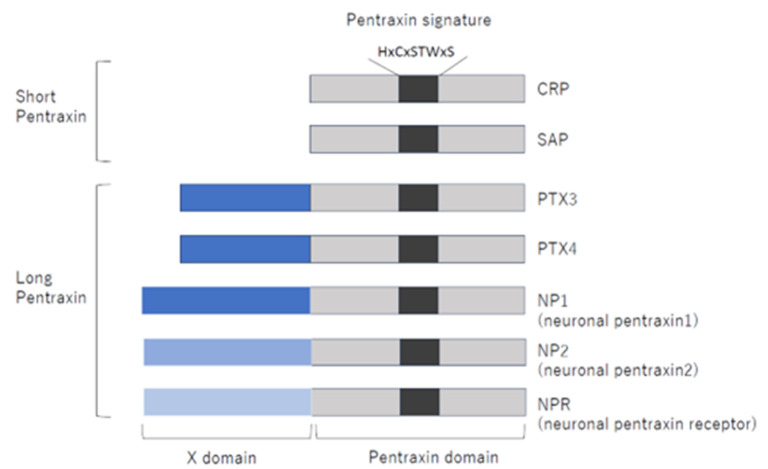
**The Structure of Pentraxin Family.** Schematic presentation of the structure of pentraxin family proteins. For details, please see the explanation in the text.

CRP is a protein belonging to the pentraxin family. The pentraxin family consists of several proteins that form a superfamily (Figure 2) [23,24]. Pentraxin superfamily possess a highly conserved pentraxin domain consisting of approximately 200 amino acid residues at their C-terminus. In particular, a sequence consisting of eight amino acids (HxCxS/TWxS, where x can be any amino acid) is conserved and is referred to as the pentraxin signature. The length of the N-terminal region adjacent to the pentraxin domain varies, and these proteins are classified into the short pentraxin family and the long pentraxin family. CRP and serum amyloid P component (SAP) are classified as short pentraxins. PTX3 (pentraxin 3), which is attracting attention as a biomarker for cardiovascular events [25,26], is classified as a long pentraxin. Human SAP has been identified as a protein that shares 51% amino acid sequence homology with CRP; however, whereas CRP functions as an acute-phase protein in humans, SAP is constitutively expressed in humans. In mice, on the other hand, SAP functions as an acute-phase protein and is considered to be the mouse equivalent of human CRP. CRP is considered a key humoral immune protein that functions in innate immune processes such as complement activation, neutrophil activation, and the removal of dead cells; the fact that sap-/- mice develop conditions resembling glomerulonephritis and SLE [27] clearly demonstrates its biological significance. While human CRP is primarily produced in the liver via IL-6, PTX3—a member of the long pentraxin family—is produced directly by vascular tissues and immune cells via IL-1β and TNF-α, making it a promising biomarker for cardiovascular events [25,26]. Although small amount, it has been reported that CRP is produced by macrophages, vascular endothelium, endothelial cells, adipocytes, peripheral blood mononuclear cells, and renal cells [28,29,30,31,32,33,34,35].

## 4. Induction of CRP Production

**Figure 3:**
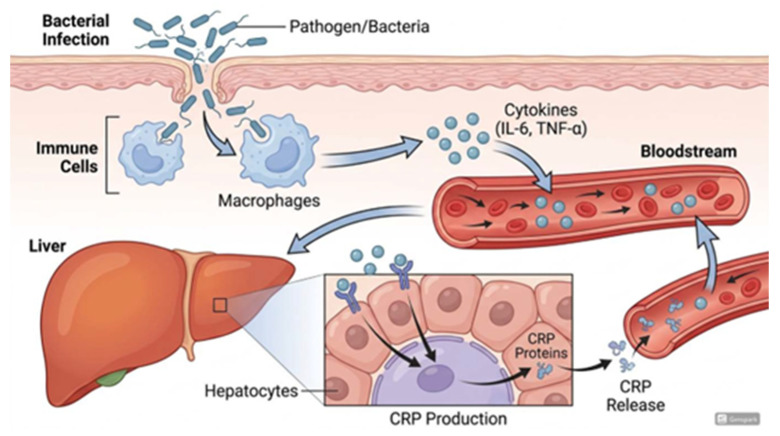
**Schematic illustration of CRP production against bacterial infection.** When a host contracts a bacterial infection, immune cells respond, leading to the local production of inflammatory cytokines, which act on hepatocytes via the bloodstream, resulting in the production of CRP. For details, please see the explanation in the text.

Regarding the mechanism of CRP production in humans, monocytes and macrophages are primarily activated in response to “pathogenic microorganisms” or “tissue injury,” leading to the production of inflammatory cytokines such as TNF-α and IL-1β. These cytokines act on Kupffer cells in the liver, inducing IL-6 production, which ultimately results in the synthesis and secretion of CRP by hepatocytes (Figure 3). In hepatocytes, CRP gene expression is primarily induced by IL-6 [36]. This is mediated by STAT3 and C/EBP [37,38,39,40]. It is reported that IL-6 alone is insufficient to induce enough CRP gene expression [41,42]. IL-1 is reported to have a synergistic effect with IL-6 in enhancing CRP gene expression [36], and this effect is thought to be mediated by MAPK and NF-κB [42]. IL-1 and TNF-α also induce CRP weakly [39]. It has been reported that IL-17 induces CRP gene expression via the MAPK-NF-κB pathway in an IL-1- and IL-6-independent manner, suggesting the importance of IL-17 in chronic inflammation [43]. Baseline serum CRP levels vary among individuals and are said to be influenced by factors such as age, gender, smoking, obesity, and blood pressure [44] as well as genetic polymorphisms [45]. Furthermore, it has been reported that polymorphisms in the intron region of the CRP gene influence interindividual variation, with 35–40% or 40–62% of this variation attributed to genetic factors [46,47]. It has also been reported that polymorphisms in the IL-6 gene influence baseline CRP levels [48]. Until now, no mutations or deletions in the alleles have been reported [44]. In bacterial infections, serum CRP levels are said to begin rising within 12 to 24 h, peak between 24 and 72 h, and rise from 0.1 mg/dL to over 50 mg/dL in severe infections [49]. According to a study examining the plasma half-life of CRP using I-125-labeled CRP in healthy subjects, the plasma half-life of CRP was reported to be 19 h [50]. Similarly, regarding TNF-α, IL-6, and PCT—which are also important biomarkers of acute inflammation—a study that administered E. coli-derived endotoxin to healthy subjects to examine their blood dynamics found that TNF-α rose 1 h after administration, peaked at 90 min, and returned to pre-administration levels after 6 h; IL-6 began to rise slightly later, peaked after 3 h, and returned to pre-administration levels after 8 h; and PCT became detectable after 4 h, peaked after 6 h, and remained elevated for 8 to 24 h [51]. On the other hand, CRP is constantly produced in the body, although at low levels. Highly sensitive CRP (hs-CRP) tests have been developed to measure changes in CRP levels as low as approximately 0.01 mg/dL, and in recent years, attention has been drawn to the fact that these measurements (hs-CRP) are useful as biomarkers reflecting the risk of long-term cardiovascular complications and diabetic complications [52,53,54,55].

## 5. Biological Function of CRP

### 5.1. The Effect of CRP on Neutrophils

It has been demonstrated that CRP promotes bacterial phagocytosis by neutrophils through its recognition of and binding to PC on the bacterial surface, because neutrophils do not phagocytose C-polysaccharide (CPS) on its own, but do so in a CRP-dependent manner [56,57,58,59]. This effect is thought to be mediated by the complement system [60,61,62,63]. On the other hand, the effect of CRP on neutrophil migration was complicated. At low concentrations (0.01–0.1 mg/dL), it promotes neutrophil migration, but no such effect was observed at high concentrations (6–12 mg/dL) [64]. Furthermore, CRP concentrations of 2.5 mg/dL or higher exhibited a concentration-dependent inhibitory effect on chemotactic stimuli, completely inhibiting them at a concentration of 10 mg/dL. Phorbol 12-myristate 13-acetate (PMA) activates neutrophils and promotes the production of reactive oxygen species. However, while CRP exhibited a synergistic effect when CRP was <0.5 mg/dL, it exhibited an inhibitory effect when CRP was >1 mg/dL [64]. Neutrophil activation by platelet-activating factor (PAF) was also inhibited by high concentrations of CRP (>10 mg/dL) [65]. It has been reported that high levels of CRP (>1 mg/dL) increase intracellular cAMP and inhibit neutrophil activation [66,67]. As our understanding of the structural changes in CRP has deepened, the reasons behind the complex phenomena described above have gradually become clear. When recombinant mCRP is added to neutrophils, NF-κB and activator protein-1 (AP-1) activation, as well as IL-8 production, are induced within 4 h, and this is 60–70% inhibited by the addition of anti-CD16. In contrast, no response was observed 4 h after the addition of pCRP, but IL-8 production was observed 24 h later [68]. In an acute kidney injury model, administration of pCRP exacerbated kidney injury; however, this exacerbation was suppressed by administration of 1,6-bis(phosphocholine)-hexane (1,6-bisPC), which inhibits the conversion of pCRP to mCRP [69]. Taking all of the above phenomena into account, it is considered that when pCRP binds to bacteria or damaged cells, a transformation occurs from pCRP → pCRP* → mCRP, and that mCRP induces the migration and activation of neutrophils, thereby stimulating their phagocytic activity. mCRP possesses an epitope that binds to the neutrophil FcγRIIIa/b (CD16) [9], and this, along with hydrophobic interactions, enhances its binding to neutrophils. On the other hand, pCRP binds to the FcγRI (CD64) and FcγRIIa (CD32) receptors on neutrophils [70,71]. The binding of mCRP to neutrophils is thought to reach near saturation at mCRP levels below 0.5 mg/dL. In other words, the CRP concentration normally present in serum is sufficient for neutrophil activity during the early stages of inflammation. On the other hand, high concentrations of pCRP are thought to exert an anti-inflammatory effect.

### 5.2. The Effects of CRP on Macrophages and Monocytes

In the 1980s, it was reported that CRP increases the antitumor activity of macrophages [72]. Subsequently, it was reported that CRP promotes the secretion of IL-1, IL-6, and TNF-α from monocytes [73]. Secretion of TNF-α peaks 4 to 8 h after stimulation, while that of IL-1 and IL-6 peaks 16 h later. Furthermore, adding CRP to the monocyte cell line THP-1 enhanced the expression of CCR2 (CC chemokine receptor 2), which was inhibited by anti-FcγRI (CD64) antibodies [74]. Furthermore, it has been reported that CRP induces tissue factor (TF) expression from monocytes after 4 h [75]. These findings suggest that pCRP directly stimulates macrophages. It has been shown that recombinant mCRP increases inducible nitric oxide synthase (iNOS) production in monocytes, whereas pCRP, conversely, reduces NO/iNOS production [76]. When iNOS is induced in macrophages, large amounts of NO are produced, which directly kills pathogens such as bacteria, viruses, and parasites. It has also been reported that CRP enhances the expression of liver X receptor (LXR)α and exerts anti-inflammatory effects [77]. LXR agonists suppresses the production of inflammatory cytokines in macrophages [78,79].

### 5.3. Effects of CRP on Vascular Endothelial Cells

Early studies examining the effects of CRP on vascular endothelial cells showed that culturing these cells in the presence of CRP for 24 h increased the expression of ICAM-1 and VCAM-1 [80] and suppressed endothelial nitric oxide synthase (eNOS) activity [81,82]. These effects promote the infiltration of inflammatory cells, cause vasoconstriction, and contribute to the progression of inflammation. Subsequent studies have shown that the pro-inflammatory effects of CRP are mediated by mCRP. mCRP stimulation occurs via p-38 MAP kinase and is inhibited by the p-38 MAPK inhibitor SB203580. Furthermore, this effect is partially inhibited by anti-FcγRIII (CD16) antibodies but is not affected by anti-FcγRII (CD32) antibodies [83]. It has been shown that lipid rafts play a crucial role in the binding of mCRP to vascular endothelial cells, and that this binding depends on the aa35–47 region (a putative cholesterol-binding consensus sequence) and the C-terminal aa199–206 region of mCRP. It is believed that the partial inhibition caused by anti-CD16 antibodies is due to the reasons mentioned above. Disruption of lipid rafts by using methyl-β-cyclodextrin or nystatin inhibits the production of MCP-1 and IL-8 from vascular endothelial cells, as well as the expression of ICAM-1, VCAM-1, and E-selectin, in response to mCRP stimulation [8].

### 5.4. Monomeric CRP Causes Platelet Aggregation and Activation

It has been reported that heat-treated, acid-treated, or chelated CRP causes platelet aggregation and activation [84,85,86]. As mentioned earlier, when subjected to heat treatment, acid treatment, or chelation, pCRP undergoes structural changes and ultimately dissociates into mCRP. On the other hand, pCRP is believed to inhibit platelet aggregation [87]. Based on the above, it is thought that the conversion of pCRP to mCRP induces platelet aggregation and activation. Subsequent studies using recombinant mCRP demonstrated that, under shear stress conditions, mCRP activates prothrombotic activity, whereas pCRP inhibits it [88].

### 5.5. Complement Activation by CRP and Its Regulation

The fact that the addition of CPS to plasma containing pCRP results in complement consumption indicates that the CPS-CRP complex plays a crucial role in the complement-mediated innate immune response [89,90]. Complement activation has also been observed in complexes involving CRP and polycations, positively charged liposomes [91], and DNA [92]. CRP binds to C1q and activates the classical complement pathway. In the subsequent reaction, C1, C4, and C2 are consumed, but only a portion of C3 is consumed, and activation of the terminal complement components C5–9 occurs to a very limited extent [63,90,93]. This indicates that complement activation by CRP does not result in lytic effects. When pCRP binds to PC on the cell membrane, a structural change occurs, resulting in the formation of mCRP. It has been demonstrated that recombinant mCRP binds to the collagen-like domain of C1q [94]. Interestingly, mCRP alone binds to C1q but acts as an inhibitor of subsequent complement activation. In contrast, mCRP bound to oxidized LDL (low-density lipoprotein) binds to C1q and activates the classical complement pathway. CRP suppresses complement system amplification, the alternative pathway, and the terminal pathway by reducing the convertase activity of C3 and C5 [95]. This action is thought to be Factor H-dependent [62,96]. Since the binding of Factor H to CRP is not affected by EDTA or PC, it is thought to be mediated by pCRP [62]. Factor H is an inhibitor of the complement pathway. When Factor H binds to C3b, it promotes the inactivation of C3bBb or C3bBbP—the C3 convertases of the alternative pathway—and regulates the C5 convertase by competitively inhibiting the binding of C3b to C5. Properdin binds to the surface of bacterial and cellular membranes, stabilizes C3 and C5 convertases, and activates the complement pathway. By binding to properdin, mCRP inhibits its binding to cell membranes, thereby suppressing the terminal complement pathway [97]. pCRP does not bind to properdin.

Based on the above, while both pCRP and mCRP are involved in the initial local inflammatory response, it can be considered that an increase in pCRP plays a regulatory role in suppressing the spread of the inflammatory response and limiting tissue damage.

## 6. The Association Between CRP Gene Polymorphisms and Diseases

It has been reported that individuals with CRP levels of 0.3 mg/dL or higher have a 1.6-fold and 1.3-fold higher risk of ischemic heart disease and ischemic cerebrovascular disease, respectively, compared to those with levels below 0.1 mg/dL [52]. As mentioned earlier, baseline CRP levels are influenced by genetic factors [45] as well as age, gender, smoking, obesity, and hypertension [44]. Numerous studies have been reported on the relationship between chronic diseases and CRP genetic polymorphisms. Nine SNPs that affect CRP levels have been reported (Table 1) [98]. Four are located in 5′-upstream regions (rs3116653, rs3116654, rs3122012, rs3091244), one is located within the intron (rs1417938), and the remaining four are located in the 3′-untranslated (rs1130864) and the 3′-flanking regions (rs1205, rs3093077, rs12029262). Although rs1800947 (G > C), a synonymous SNP in exon 2, showed no statistical significance (*p* = 0.09), there are reports suggesting that it influences CRP levels [99].

It has been demonstrated that the rs3091244 polymorphism (C > A > T) in the CRP gene promoter region is associated with the development of abdominal aortic aneurysms (AAA) [100]. Compared to the common CC genotype, the risk of AAA was 4.88 times higher (95% confidence interval [CI], 2.96–8.04) for the rare genotypes TT, AA, and TA, and 2.38 times higher (95% CI, 1.69–3.36) for the heterozygous genotypes CT and CA.

Concerning the rs1205 polymorphism (C > T) in CRP gene, CT genotype (odds ratio (OR) = 1.799) and T allele (OR = 1.733) are associated with increased risk of type 1 Diabetes mellitus (T1D), while CC genotype decreases the risk of this condition (OR = 0.458) [101]. In rs1800947 (G > C) single-nucleotide polymorphism (SNP) within exon 2 of the CRP gene, homozygous CC genotypes and heterozygous GC had higher risk for T2DM compared to the GG genotype at odds ratio of 20.56 (1.16–362.1) and 2.72 (1.12–6.61), respectively [102]. In another study, homozygous CC genotypes of rs3093059 (T > C) have been reported to have higher risk (recessive: OR, 7.01; 95% CI, 1.16–42.22) for T2DM in the white population [103].

It has also been reported that rs1800947 (G > C) CRP gene SNP is associated with increased risk for acute myocardial infarction (AMI) (OR = 3.86) [104]. Another study revealed that rs1800947 (G > C) gene variation influenced plasma CRP levels, but was not associated with the risk for AMI [99].

In Japanese cohort, it has been reported that CRP polymorphisms (rs3093059 (T > C) and rs1205 (C > T)) were not associated with the susceptibility to ischemic stroke (IS) [105]. Meta-analysis of eligible 22 related articles concluded that the CRP polymorphisms (rs3093059 (T > C) and rs1205 (C > T)) were not associated with the susceptibility to IS [106]. The rs2794521 (A > G) polymorphism was not significantly associated with the risk of IS from meta-analysis study [107]. On the other hand, carriers of the rs2794521(GG) genotype had a significantly higher CRP level on the first day after IS versus heterozygotes (*p* = 0.023). The improvement in neurological state evaluated with the National Institutes of Health Stroke Scale (NIHSS) was significantly better in rs2794521 (AA) patients in comparison with other genotypes (*p* = 0.035) [108].

Concerning with cancer (endometrial cancer, lung cancer and colorectal cancer), meta-analysis have shown that CC genotype in rs1800947 (G > C) polymorphisms have an almost 4-fold higher risk of cancer than those with the GG or GC and GG genotypes. In subgroup analysis, rs1205 (C > T) polymorphism showed increased colorectal cancer risk in the TT genotype (TT vs. CC: OR  =  1.15, 95% CI  =  1.01–1.31; TT vs. CT  +  CC; OR  =  1.17, 95% CI  =  1.03–1.32) [109]. In a Chinese cohort, the TT genotype was significantly associated with 2.48-fold increased risk of lung cancer in comparison with CC genotype. The TT genotype was also related with increased lymphatic metastasis (TT vs. CC, OR 1.88, 95% CI, 1.03–3.45) [110]. It has also been reported that TT genotype had a significantly greater likelihood of developing lymph node metastasis in submucosal thoracic esophageal squamous cell carcinoma [111], submucosal invasive gastric cancer [112] and invasive breast cancer [113].

A cross-sectional investigation of almost 2700 individuals from an urban population reported that a CRP gene polymorphism, rs1130864 (C > T), was associated both with increased amounts of CRP, as well as an elevated risk for developing Post-Traumatic Stress Disorder (PTSD) symptoms and increased fear learning. ‘CC’ genotype had an average PTSD Symptom Scale (PSS) value of 14.1, whereas those with one or two ‘T’ alleles had an average PSS value of 15.7. Additionally, individuals with the ‘CC’ genotype had lower serum CRP compared to those carrying the ‘T’ allele (*p* < 0.005) [114].

In studies focusing on children—a population in which the period of environmental influence is relatively short and genetic factors are likely to play a significant role—there are reports suggesting that the rs1205 polymorphism is associated with obesity and metabolic disorders [115,116].

However, as is commonly mentioned, the relationship between genetic polymorphisms and disease susceptibility involves complex interactions with numerous other genetic polymorphisms and various environmental factors; therefore, caution is required when interpreting these findings.

## 7. Future Outlook of Targeted Therapy for CRP

There are several attempts that have been made to develop molecularly targeted therapies for CRP.

One approach involves the use of antisense technology, which has been shown to reduce CRP levels in mice, rats, and humans [117,118,119]. However, although a decrease in CRP levels was observed, no clinical benefit was observed in patients with rheumatoid arthritis [120].

Another approach involves the use of small molecules that inhibit the binding of CRP to PC. The bivalent compound 1,6-bis(phosphocholine)-hexane (bis-PC) binds two pCRP molecules to form a decameric complex, thereby inhibiting the binding of pCRP to the site of inflammation [69]. However, concerns that the formed polymers may cause adverse reactions as immune complexes via FcγR have prevented their clinical application. The monovalent compound C10M [3-(dibutylamino)propyl) phosphonic acid] binds to the phosphocholine binding pocket of pCRP and prevents pCRP binding to PC residues exposed on the surface of activated or damaged cells and subsequently the formation of pCRP*/mCRP [121]. Importantly, it was demonstrated that C10M preserves the opsonic effect of pCRP, thereby retaining antibacterial host defense activity.

## 8. Conclusions

It has been approximately 100 years since the discovery of CRP. During that time, it has long been widely used in clinical medicine as a biomarker of acute inflammatory responses. In recent years, as its molecular mechanisms of action have been elucidated, it has regained attention not only as a key humoral factor in acute inflammation but also in chronic inflammation, and has become a target for new molecular therapies. In particular, the notion that the CRP levels present at baseline are sufficient to induce an initial inflammatory response, and that the subsequent surge in CRP—which occurs as an acute response—is involved in immune regulation, is a key point in understanding the biological process of inflammation. This concept is crucial for understanding the pathophysiology of so-called “chronic inflammatory diseases”—such as cancer, cardiovascular disease, neurodegenerative diseases, and metabolic disorders—which have become increasingly significant in public health in recent years, and for developing strategies to address them. With a deeper understanding of the newly identified biological functions of CRP that have come to light in recent years, and by reexamining the pathophysiology of acute and chronic inflammation from a different perspective, new horizons may open up in medical science.

## Figures and Tables

**Table 1:** Association of individual CRP gene polymorphism that affects CRP level.

Allelic Variant	Variant Type	Major > Minor Allele	Minor Allele Frequency	*p*
rs3116653	5′-Upstream	C > G	0.31	0.0008
rs3116654	5′-Upstream	T > C	0.12	0.04
rs2794517	5′-Upstream	C > T	0.3	0.99
rs3122012	5′-Upstream	A > G	0.31	0.001
rs3091244	5′-Upstream	C > T	0.31	<0.0001
		C > A	0.07	
rs1417938	Intron	A > T	0.31	0.001
rs1800947	Synonymous, coding	G > C	0.06	0.09
rs1130864	3′-Untranslated	C > T	0.31	0.0006
rs1205	3′-Flanking	G > A	0.33	<0.0001
rs2808630	3′-Flanking	A > G	0.28	0.43
rs3093077	3′-Flanking	T > G	0.07	0.002
rs12029262	3′-Flanking	C > G	0.09	0.002
rs876538	3′-Flanking	G > A	0.2	0.76

This table is reproduced and modified with permission from Kathiresan S. et al., Circulation; published by AHA Journals, 2006 [98].

## Data Availability

No new data were created or analyzed in this study.
